# Compartment-specific immune responses to BoAHV-1 transplacental infection in late gestation

**DOI:** 10.3389/fvets.2026.1845115

**Published:** 2026-06-23

**Authors:** Felipe Cheuquepán, Irene Agulló-Ros, María A. Risalde, Mercedes M. Burucúa, Silvina Quintana, Natalia Plá, Sandra E. Pérez, María Valeria Scioli, Germán J. Cantón, Dadin P. Moore, Anselmo C. Odeón, Maia S. Marin, Eleonora L. Morrell

**Affiliations:** 1 Consejo Nacional de Investigaciones Científicas y Técnicas (CONICET), Buenos Aires, Argentina; 2 Departamento de Biología, Facultad de Ciencias Exactas y Naturales (FCEyN), Universidad Nacional de Mar del Plata (UNMDP), Mar del Plata, Argentina; 3 Departamento de Anatomía y Anatomía Patológica Comparadas y Toxicología, Grupo de Investigación GISAZ, UIC Zoonosis y Enfermedades Emergentes ENZOEM, Universidad de Córdoba, Córdoba, Spain; 4 Instituto de Investigaciones de Producción, Sanidad y Ambiente (IIPROSAM), FCEyN, UNMDP-CONICET, Mar del Plata, Argentina; 5 Instituto de Biología Molecular Aplicada, Mar del Plata, Argentina; 6 Centro de Investigaciones Veterinarias de Tandil (CIVETAN) - CONICET, Facultad de Ciencias Veterinarias, UNCPBA, Tandil, Argentina; 7 Instituto de Innovación para la Producción Agropecuaria y el Desarrollo Sostenible (IPADS, INTA-CONICET), Balcarce, Argentina; 8 Facultad de Ciencias Agrarias, UNMDP, Balcarce, Argentina

**Keywords:** BoAHV-1, bovine placenta, innate immune response, maternal-fetal interface, toll-like receptors, transplacental infection

## Abstract

*Varicellovirus bovinealpha1* (BoAHV-1) is an important cause of reproductive failure in cattle and can induce abortion following transplacental infection during late gestation. However, immune-associated responses at the conceptus (placenta and fetus) remain incompletely understood. This study aimed to characterize maternal and fetal immune-associated responses to BoAHV-1 vertical infection by evaluating the expression of endosomal Toll-like receptors (*TLR3*, *TLR7* and *TLR9*), cytokines, and the antimicrobial peptide *BMAP28*, together with the distribution of immune-cell markers in placental and fetal tissues. Pregnant dams (infected, *n =* 7; controls, *n =* 4) were experimentally infected with BoAHV-1 during late gestation and euthanized at 15 days post-infection (dpi). Peripheral blood leukocytes (PBLs) were collected at 3 and 10 dpi, and placental and fetal tissue samples were collected during necropsy. Relative mRNA expression of innate immune genes was assessed by RT-qPCR, while immune-cell markers were characterized by immunohistochemistry. Maternal tissues, including PBLs and placental caruncles, showed reduced expression of *TLR9* and *TNF-α*. Fetal lungs showed increased expression of endosomal TLRs, *TNF-α*, *IL-12* and *BMAP28*, accompanied by increase of CD3^+^ cells and iNOS-associated cells. The fetal spleen showed specific changes in gene expression, whereas the fetal liver presented a distinct transcriptional pattern with increased TLR expression and reduced cytokine transcript levels. Overall, BoAHV-1 transplacental late infection was associated with distinct patterns of immune-related gene expression and immune-cell marker distribution across maternal, placental and fetal tissues, providing an integrated view of immune-associated responses at day 15 post-infection.

## 1 Introduction

Var*icellovirus bovinealpha1* (BoAHV-1) is a globally distributed pathogen that establishes lifelong latent infections with the potential for periodic reactivation (1). This virus causes a wide range of clinical manifestations in cattle, including respiratory and genital disease, and may also undergo systemic dissemination, particularly in susceptible animals, allowing the virus to reach the reproductive tract and cross the placenta (2). Consequently, BoAHV-1 is recognized as an important cause of late-gestation abortion in cattle (3), although the interval between maternal infection and fetal infection or abortion is variable, ranging from a few days to several weeks or longer (2, 41).

During late gestation in cattle, the fetal immune system is relatively developed, reflecting its progressive maturation during gestation, and the fetus is capable of mounting innate and adaptive immune responses (4, 5). The endometrium of pregnant ruminants contains specialized structures termed placentomes characterized by the presence of interdigitated fetal villi (cotyledons) and maternal villi (caruncles), which increase the surface for maternal-fetal exchange (6). Additionally, the bovine placenta maintains a tightly regulated immune environment to allow fetal tolerance while protecting against pathogens (7). Toll-like receptors (TLRs), particularly endosomal TLRs (*TLR3*, *TLR7*-*9*), are critical components of innate immunity that recognize viral nucleic acids and activate antiviral responses (8), including the induction of antimicrobial peptides such as bovine cathelicidin *BMAP28* (9). Existing research in non-pregnant experimentally infected cattle has established the involvement of TLRs in the response to BoAHV in peripheral blood leukocytes (PBL) (10), as well as nervous (11, 12) and respiratory (13) tissues. However, their specific involvement at the maternal-fetal interface during late-gestational BoAHV-1 infection remains unclear. Although TLR activation is essential for antiviral defense, excessive or dysregulated responses may be associated with inflammatory processes that could affect placental function (7, 14).

Macrophages also play a central role in regulating the immune response at the maternal-fetal interface in cattle, where they contribute to tissue homeostasis, immune tolerance and defense against pathogens (15). This balance becomes particularly relevant during viral infections, including BoAHV-1 infection, as macrophages are among the first innate immune cells to recognize viral components through pattern recognition receptors such as TLRs, triggering their activation and the rapid production of type I interferons, cytokines and chemokines that contribute to early viral control and recruitment of additional immune cells (16). However, several alphaherpesviruses have been reported to interact with innate signaling pathways (17), potentially limiting macrophage antiviral functions and altering their activation profile during infection (18). Macrophages exhibit functional plasticity and can express markers associated with pro-inflammatory or anti-inflammatory-related profiles depending on the local microenvironment (19). Markers such as iNOS and CD163 are commonly used to characterize macrophage-associated functional profiles, although they do not define discrete activation states *in vivo* (20). In cattle, increased representation of macrophages expressing anti-inflammatory-associated markers has been reported during the second half of gestation (21), suggesting a role in maintaining maternal-fetal tolerance. However, how these immune-cell populations are distributed during BoAHV-1 transplacental infection remains unclear.

Although existing research has characterized several aspects of fetal immune development (4, 5) and tissue-specific immune responses during transplacental infection, including BoAHV-1 infection (22), these studies remain limited. Therefore, this study aimed to characterize immune-associated responses across maternal, placental and fetal compartments following experimental BoAHV-1 transplacental infection. Specifically, we analyzed the relative gene expression of endosomal TLRs, selected cytokines and *BMAP28*, along with the distribution of immune-cell markers at the maternal-fetal interface and in fetal tissues from experimentally infected pregnant dams. We hypothesized that BoAHV-1 infection is associated with tissue-specific differences in immune-related gene expression and immune-cell marker distribution across these compartments.

## 2 Materials and methods

### 2.1 Animals and experimental design

The experimental design and tissue sample collection methods have been reported previously (22). The present study utilized PBL samples to assess the innate immune response in the periphery. In brief, 11 healthy, pregnant Aberdeen Angus heifers, approximately 5 years old, from National Institute of Agricultural Technology (INTA) Balcarce, Argentina, were selected for the study. Pregnancy was confirmed by ultrasonography 60 days after estrus synchronization and natural mating. All animals were screened and found seronegative for major bovine pathogens (*Neospora caninum*, *Leptospira* spp., BVDV, and BoAHV-1) and free of abortifacient venereal agents (*Campylobacter fetus* and *Tritrichomonas foetus*). At 270 days of gestation, the pregnant dams were randomly assigned into two groups: Group 1 (*n =* 7) animals were intravenously challenged with 2 mL of BoAHV-1 Cooper strain (1 × 10^5.87^ TCID_50_/ml) (22, 23), and Group 2 (*n =* 4), the control group, animals were intravenously inoculated with 2 mL of sterile phosphate-buffered saline (PBS). Blood from each dam was collected on 3 and 10 days post-infection (dpi) for PBLs isolation as previously described (10). Animals were monitored daily by veterinary personnel throughout the experimental period, including clinical status, indicators of impending parturition and animal welfare parameters. Humane endpoints established by the Institutional Animal Ethics Committee were followed during the study. Pregnancy status was confirmed by ultrasonographic examination prior to necropsy. At day 15 post-infection, the dams were euthanized according to the protocol approved by the Animal Ethics Committee at INTA Balcarce (CICUAE#187/2019). All fetal organs and the placenta of each dam were immediately removed and examined following standard gross pathology procedures (24). Placental samples were obtained from placentomes, which are the functional units of the ruminant placenta composed of the fetal cotyledon and the maternal caruncle. Cotyledons correspond to the fetal component, whereas caruncles represent the maternal endometrial tissue. This distinction was considered for the interpretation of tissue-specific immune responses. Tissue samples were collected and fixed in 10% buffered formalin for 24 h for further histopathological and immunohistochemical studies. In addition, placentomes and fetal tissue samples were stored at − 80 °C immediately after collection and maintained at this temperature until processing for quantitative polymerase chain reaction (qPCR) and reverse transcription qPCR (RT-qPCR).

### 2.2 Clinical signs and confirmation of BoAHV-1 infection

All dams were monitored for rectal temperature, the presence of nasal and ocular discharges, inappetence, lethargy, or any other clinical signs. Blood samples were taken at 0, 3, 7, 10 and 14 dpi for serum neutralization assays. Additionally, whole blood samples collected at 3 and 10 dpi were used for PBL isolation Neutralizing antibodies to BoAHV-1 in sera were determined by a serum neutralization test using Madin-Darby bovine kidney (MDBK) cells, and detection of BoAHV-1 DNA in placentomes and fetal tissues (lung, spleen and liver) by qPCR, as previously reported by Burucúa et al. (22).

### 2.3 Histology

Samples of brain, heart, lung, liver, spleen, kidney, striated muscle, tongue, adrenal gland, colon and small intestine from fetuses from Group A (BoAHV-1 infected) and two randomly selected fetuses from Group B (control) were examined for microscopic lesions. Additionally, five placentomes and five intercotiledonary chorion from each corresponding dam were ramdomly selected. Tissue samples were fixed in 10% buffered formalin for 24 h, dehydrated in a graded series of ethanol, immersed in xylol and embedded in paraffin wax using an automatic processor. Sections of each tissue block were cut at 3 μm, mounted on glass microscope slides and stained with hematoxylin and eosin (H&E). All samples were observed under an optical microscope (Nikon, eclipse E 200).

### 2.4 Expression analysis of TLR, cytokine and cathelicidin genes in maternal and fetal tissues and PBLs

Total RNA was isolated from each fetal bovine tissue (lung, liver, spleen, cotyledon), maternal samples (caruncle and PBL samples collected at 3 and 10 dpi) and placentomes of all dams and fetuses from both groups (infected, *n =* 7; controls, *n =* 4) using RNeasy Mini Kit (Qiagen Inc., Valencia, CA, USA) according to the manufacturer’s protocol and digested with RQ1 RNase- Free DNase (Promega, Madison, WI, USA) for 30 min at 37 °C to remove genomic DNA (gDNA). RNA quality and quantity were determined using an Epoch Microplate Spectrophotometer (BioTeK, Winooski, VT, USA). Complementary DNA (cDNA) was prepared from 1 μg of total RNA using Moloney murine leukemia virus reverse transcriptase (SuperScript III Reverse Transcriptase, Thermo Fisher Scientific, USA). Negative controls, omitting RNA or reverse transcriptase, were included. RT-qPCR reactions for bovine genes of immunological receptors and mediators (*TLR3*, *TLR7*, *TLR9*, *TNF-α*, *IFN-β1*, *IL-12p40*, *BMAP28*) were carried out using the specific primers described in Table 1. Expression of the housekeeping gene glyceraldehyde-3-phosphate dehydrogenase (*GAPDH*) was used as an internal control (25). qPCR reactions contained specific forward and reverse primers (800 nM), PCR Master Mix (1X, FastStart Universal SYBR Green Master Rox, Roche, Mannheim, Germany) and cDNA (1 μL) in a final volume of 20 μL. Amplification and detection of the specific products were carried out in an Applied Biosystems 7,500 cycler, with the following amplification conditions: 2 min at 50 °C, 10 min at 95 °C, and 40 cycles of 20 s at 95 °C and 60 s at 60 °C. After amplification, a melting curve analysis was performed, which resulted in a single product-specific melting curve. Samples were run in duplicates and negative controls for cDNA synthesis and PCR procedures were included. The amplification efficiency was determined for each gene using 10-fold dilutions of the cDNA. *GAPDH* expression levels remained constant in samples from all animals and a linear relationship between the amount of the template and Ct values was observed when the amplification efficiency for each gene was determined (data not shown). Results are reported as the mean fold change of gene transcription levels (TLRs, cytokines or *BMAP28*) from BoAHV-1-infected group over non-infected control group. Undetected or very low-expression values were handled according to the requirements of the Relative Expression Software Tool (REST, Qiagen Inc., Valencia, CA, USA), which incorporates such values into the analysis through its randomization-based approach. All samples were included in the analysis, and no arbitrary threshold-based exclusion criteria were applied.

**Table 1:** Sequences of primers for mRNA relative quantification by RT-qPCR.

mRNA	Primer sense	Amplicon size (base pairs)	5′ - 3′ sequences	References
*GAPDH*	F^(a)^	112	TTCTGGCAAAGTGGACATCGT	(25)
	R^(b)^		CTTGACTGTGCCGTTGAACTTG	
*BMAP28*	F	141	TCGGGAGTAACTTCGACATCACCT	(39)
	R		GGCCCACAATTCACCCAATTCTGA	
*TLR3*	F	143	CAGGTCAACAGTCCCGAA	(10)
	R		GCAGCACATTCCCCACAT	
*TLR7*	F	144	TAAAACTCTGCCCTGTGATG	(10)
	R		CCTGCTATGTGGTTAATGGT	
*TLR9*	F	113	AACCTGCCCGCCAGACCCT	(10)
	R		GCCAGGGCCACTGCCAGTG	
*TNF-α*	F	176	AGCCTCAAGTAACAAGCC	(28)
	R		TGAAGAGGACCTGTGAGT	
*IL-12p40*	F	157	AGTACACAGTGGAGTGTCAG	(40)
	R		TTCTTGGGTGGGTCTGGTTT	

^a^Forward primer. ^b^Reverse primer.

### 2.5 Immunohistochemical techniques and cell counting

To quantitatively evaluate changes in immune cell populations, the expression of the cellular markers iNOS (pro-inflammatory macrophage profile), CD163 (anti-inflammatory macrophage profile), CD3^+^ (T lymphocyte profile) and CD79α (B lymphocyte profile) was assessed by immunohistochemistry in fetal lungs (*n =* 5) and placentas (*n =* 6) from BoAHV-1 experimentally infected dams. Samples were selected based on histopathological findings. Additionally, two non-infected animals were included as controls. Briefly, sections of the formalin-fixed paraffin-embedded tissues (two placentomes, cerebrum, liver, kidney, spleen, heart and lungs of each fetus) were cut at 3 μm and processed for immunohistochemistry (IHC) using the avidin-biotin-peroxidase complex (ABC) method (26). Each section was placed in silane-coated slides [3-(triethoxysilyl)-propylamine], dewaxed and rehydrated using graded ethanol series. Endogenous peroxidase activity was blocked by incubation with 0.3% hydrogen peroxide in PBS for 30 min at room temperature (RT). The samples were subjected to different methods for antigen retrieval, depending on the primary antibody (Ab) used (Table 2). After antigen retrieval, the sections were rinsed three times in PBS (pH 7.2) for 10 min and blocked with 20% normal goat serum (Thermo Fisher Scientific) or 3% rabbit serum in PBS for 30 min at RT, for primary rabbit or goat polyclonal antibody (pAb), respectively. After this blocking stage, sections were incubated with the primary antibodies at 4 °C overnight. Then, slides were washed in PBS (three times for 5 min each) and subsequently incubated with the secondary antibodies for 30 min at RT. Primary rabbit Abs were detected using a biotinylated goat anti-rabbit IgG secondary Ab (Vector Laboratories) diluted 1:200 in PBS containing 10% normal goat serum. Similarly, primary goat Abs were detected using a biotinylated rabbit anti-goat IgG secondary Ab (Vector Laboratories) diluted 1:200 in PBS containing 1% normal rabbit serum. After three 5 min washes in PBS, samples were incubated with the ABC complex (Vectastain^®^ ABC Elite Kit, Vector Laboratories) for 1 h at RT. All tissue sections were rinsed in Tris-buffered saline and incubated with the chromogen solution (NovaRED^®^ Substrate Kit, Vector Laboratories). Finally, slides were counterstained with Harris’ haematoxylin. Bovine tissues with previously tested reactivity for the primary antibodies against the cytokines used in this study, were included as positive controls (26).

**Table 2:** Details of the primary antibodies used in the immunohistochemical study.

Specificity	Marker for	mAb/pAb	Dilution	Pre-treatment	Source
Anti-BoAHV-1	BoAHV- 1 antigen	pAb	1:1000	TC-microwave^a^	VMRD (210–70-IBR)
Anti-rabbit iNOs	Pro-inflammatory macrophages	pAb	1:100	TC-autoclave^b^	Merck Millipore
Anti-human CD163	Anti-inflammatory macrophages	mAb	1:200	TC-autoclave^b^	Bio-Rad AbD Serotec
Anti-human CD3	T lymphocytes	pAb	1:100	TC-microwave^a^	Dako/Agilent
Anti-human CD79α	B lymphocytes	mAb	1:25	TC-microwave^a^	Dako/Agilent

BoAHV-1, bovine alphaherpesvirus type 1, mAb: monoclonal antibody; pAb: policlonal antibody; TC: 0.01 M tri-sodium citrate dehydrate. ^a^Incubation with 0.1 M tri-sodium citrate dihydrate (pH 6), microwave at sub-boiling temperature for 6 min. ^b^Incubation with 0.1 M tri-sodium citrate dihydrate (pH6), autoclaved at 121 °C and 1 atm for 10 min.

Three experienced observers (I. A. R., M. A. R and E. M.) blinded to the treatment groups, performed the cell counting in 25 randomly-chosen fields of 0.2 mm^2^. Cellular identification was based on their morphological features, location and cell size. The results obtained were given as the number of positive cells per 0.2 mm^2^.

### 2.6 Statistical analysis

The relative gene expression analysis of the target genes was performed using the REST software (Qiagen Inc., Valencia, CA, USA). The REST tool compares the expression of the target gene in a sample group relative to a control group with 2–16 data points (Supplementary material), evaluating group differences for significance with a pair-wise fixed reallocation randomization test and incorporating amplification efficiency into the analysis (27). This method allows robust group-wise comparison of RT-qPCR data, particularly in studies with limited sample sizes, providing robust statistical inference without relying on assumptions of normal distribution. *p* < 0.05 were considered statistically significant. The RT-qPCR efficiency for each gene was determined by a linear regression model according to the equation: E = 10 [−1/slope]. Detailed individual expression values and measures of variability are provided in the Supplementary material to facilitate interpretation of group differences.

The results of immunohistochemical analysis were expressed as means ± standard errors (SEM) (Supplementary material). The statistical unit was the individual animal. For each animal, counts from 25 randomly selected microscopic fields were averaged and used as a single value for statistical analysis. Microscopic fields were treated as technical replicates and were not considered independent observations. Normal distribution of the data was tested with the Shapiro–Wilk test, using SPSS V18.0 statistical software (IBM Corporation, NY, USA). Statistically significant differences between means from BoAHV-1-inoculated and control animals were assessed by the Mann–Whitney nonparametric U-test (*p <* 0.05). The statistical analysis was performed with GraphPad Prism software version 8 (GraphPad Software, Inc.).

## 3 Results

### 3.1 Clinical findings and confirmation of BoAHV-1 infection

Briefly, no abortions or clinical signs were recorded in BoAHV-1-inoculated or control animals. Seroconversion was determined in BoAHV-1-inoculated dams at 14 dpi and viral transplacental infection was confirmed by BoAHV-1 DNA detection in placenta and fetal organs, as previously reported by Burucúa et al. (22). These findings on DNA detection are summarized here to provide biological context for the samples analyzed in the present study. In that previous study, viral DNA was identified in placental tissues from two fetuses, and in fetal organs including spleen (two fetuses), liver (one fetus), and lung (one fetus). One fetus tested negative for viral DNA, and all control samples were negative Burucúa et al. (22).

### 3.2 Histology

All fetuses examined in the infected group showed microscopic liver lesions characterized by mild, multifocal, focal, or periportal mononuclear hepatitis. Mild focal mononuclear placentitis was observed in tissue samples of two fetuses. Only one bovine fetus showed mild diffuse mononuclear hypercellularity in the alveolar septa and mild interstitial mononuclear myocarditis. In all cases, the mononuclear infiltrate was composed mainly of macrophages and occasional lymphocytes. In tissues from the control group there were no remarkable lesions in one fetus, and the other fetus occasionally showed mild multifocal mononuclear hypercellularity in the alveolar septa.

### 3.3 Relative mRNA expression of endosomal TLRs, cytokines and cathelicidins

#### 3.3.1 Temporal changes in TLR and cytokine expression in circulating leukocytes

**Figure 1:**
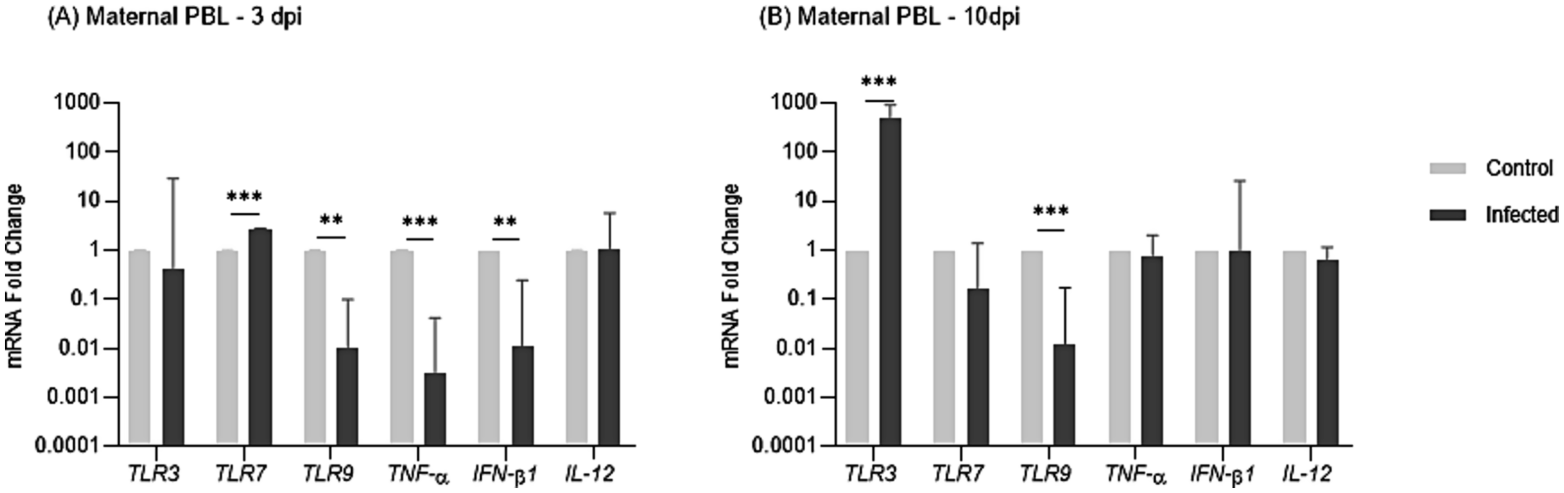
TLR and cytokine mRNA expression in maternal PBLs at 3 and 10 dpi. **(A)** 3 dpi. **(B)** 10 dpi. The bar express relative mean fold change value ± standard deviation for each mRNA gene compared against the uninfected group. Statistically significant differences are indicated by: *p < 0.05; **p < 0.01; ***p < 0.001. mRNA relative levels and statistical significance of the differences in mRNA expression were obtained with the software REST (Qiagen Inc., Valencia, CA, USA).

Analysis of immune associated transcripts in the PBLs collected from the dams revealed early and distinct changes in innate immune transcripts. At 3 dpi, infected dams exhibited distinct transcriptional changes, characterized by a significant (*p <* 0.05) up-regulation of mRNA for *TLR7* (2.66-fold), while *TLR9* (0.01-fold), *IFN-β1* (0.01-fold), and *TNF-α* (0.003-fold) showed reduced transcript levels relative to controls (Figure 1A). By 10 dpi (Figure 1B), the transcriptional landscape shifted toward a marked increase in relative *TLR3* transcript abundance (490.38-fold), although this finding should be interpreted cautiously considering the limited sample size and variability associated with low-abundance transcripts. Furthermore, a significantly (*p <* 0.05) reduced expression of *TLR9* (0.01-fold) when compared to the control group (Figure 1B) was determined. Detailed individual values and measures of variability are provided in Supplementary material.

#### 3.3.2 Organ-specific innate immune patterns in BoAHV-1-exposed fetuses

**Figure 2:**
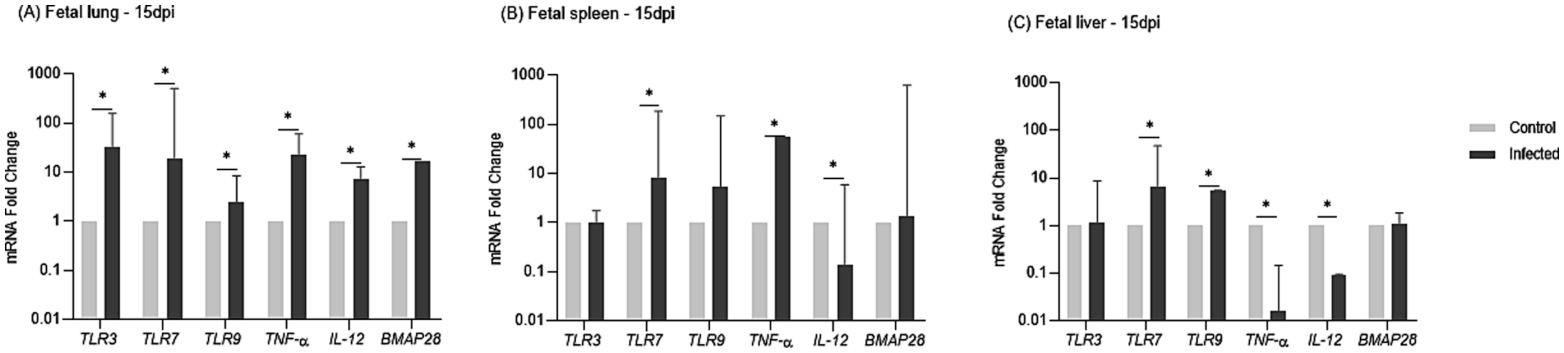
TLR and cytokine mRNA expression in fetal tissues at 15 dpi. **(A)** Fetal lungs. **(B)** Fetal spleens. **(C)** Fetal livers. The bar represents relative mean fold change value ± standard deviation for each mRNA gene compared against the uninfected group. Statistically significant differences (*p <* 0.05) are indicated by: *mRNA relative levels and statistical significance of the differences in mRNA expression were obtained with the software REST (Qiagen Inc., Valencia, CA, USA).

Infection with BoAHV-1 was associated with organ-specific transcriptional patterns in various fetal tissues (Figure 2). In the fetal lung, increased transcript levels were observed, marked by significant (*p <* 0.05) up-regulation of *TLR3* (32.26-fold), *TLR7* (19.37-fold), *TLR9* (2.50-fold), *TNF-α* (22.81-fold), *IL-12* (7.24-fold), and *BMAP28* (16.29-fold) (Figure 2A). A similar, though more focused, pattern was observed in the fetal spleen (Figure 2B), where increased mRNA levels of *TLR7* (8.14-fold) and *TNF-α* (53.97-fold) were accompanied by reduce in *IL-12* expression (0.14-fold) (Figure 2B). The fetal liver (Figure 2C) displayed a distinct transcriptional pattern. Despite significant (*p <* 0.05) up-regulation of mRNA for *TLR7* (6.32-fold) and *TLR9* (5.57-fold), expression of *TNF-α* and *IL-12* was markedly reduced (0.01 and 0.09-fold, respectively), resulting in a more restricted cytokine profile relative to the fetal lung and spleen.

#### 3.3.3 Spatial patterns and predominant reduction of innate immune transcripts at the maternal-fetal interface

**Figure 3:**
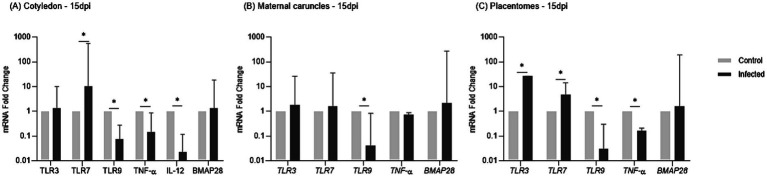
TLR and cytokine mRNA expression in maternal-fetal interface at 15 dpi. **(A)** Fetal cotyledons. **(B)** Maternal caruncles. **(C)** Placentomes. The bar expresses relative mean fold change value ± standard deviation for each mRNA gene compared against the uninfected group. Statistically significant differences (*p <* 0.05) are indicated by: *mRNA relative levels and statistical significance of the differences in mRNA expression were obtained with the software REST (Qiagen Inc., Valencia, CA, USA).

Infection with BoAHV-1 was associated with reduced transcript levels of selected innate immune markers across the fetal-maternal interface (Figure 3). In fetal cotyledons (Figure 3A), transcriptional responses were characterized by increased *TLR7* expression (10.44-fold), while *TLR9*, *TNF-α* and *IL-12* showed reduced transcript levels (0.07-, 0.14- and 0.02-fold, respectively). Maternal caruncles (Figure 3B) showed a consistent and significant (*p <* 0.05) reduction in *TLR9* expression (0.04-fold). Within placentomes, increased *TLR3* expression (26.81-fold) was observed alongside reduced *TLR9* (0.03-fold) and *TNF-α* (0.01-fold) transcript levels (Figure 3C). Reduced *TLR9* expression was observed in cotyledons, caruncles and placentomes.

### 3.4 Local immune cell response to BoAHV-1 infection

**Figure 4:**
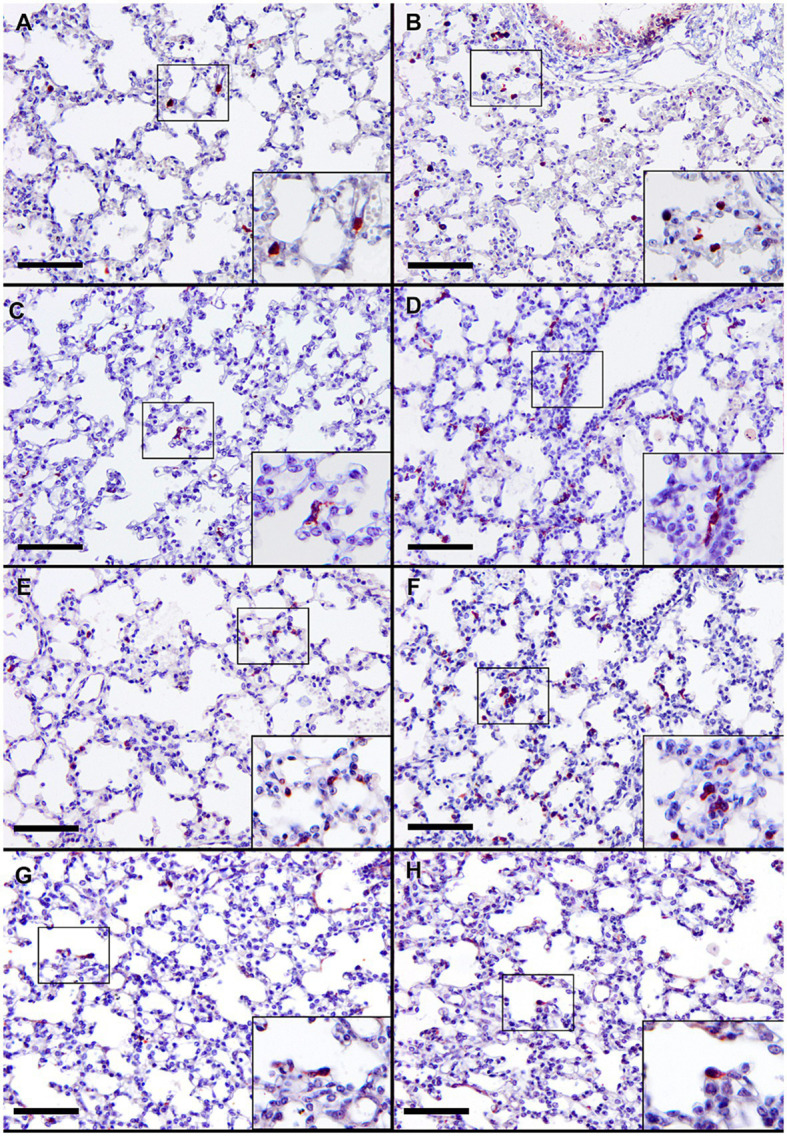
Immunohistochemical labelling in fetal lungs. **(A)** iNOS-positive cells (pro-inflammatory macrophages) in control fetuses. **(B)** iNOS-positive cells in BoAHV-1-infected fetuses. **(C)** CD163-positive cells (anti-inflammatory macrophages) in control fetuses. **(D)** CD163-positive cells in infected fetuses. **(E)** CD3-positive cells (T lymphocytes) in control fetuses. **(F)** CD3-positive cells in infected fetuses. **(G)** CD79*α*cy-positive cells (B lymphocytes) in control fetuses. **(H)** CD79αcy-positive cells in infected fetuses. Insets represent higher magnifications of the fields framed in black. Scale bar = 100 μm.

**Figure 5:**
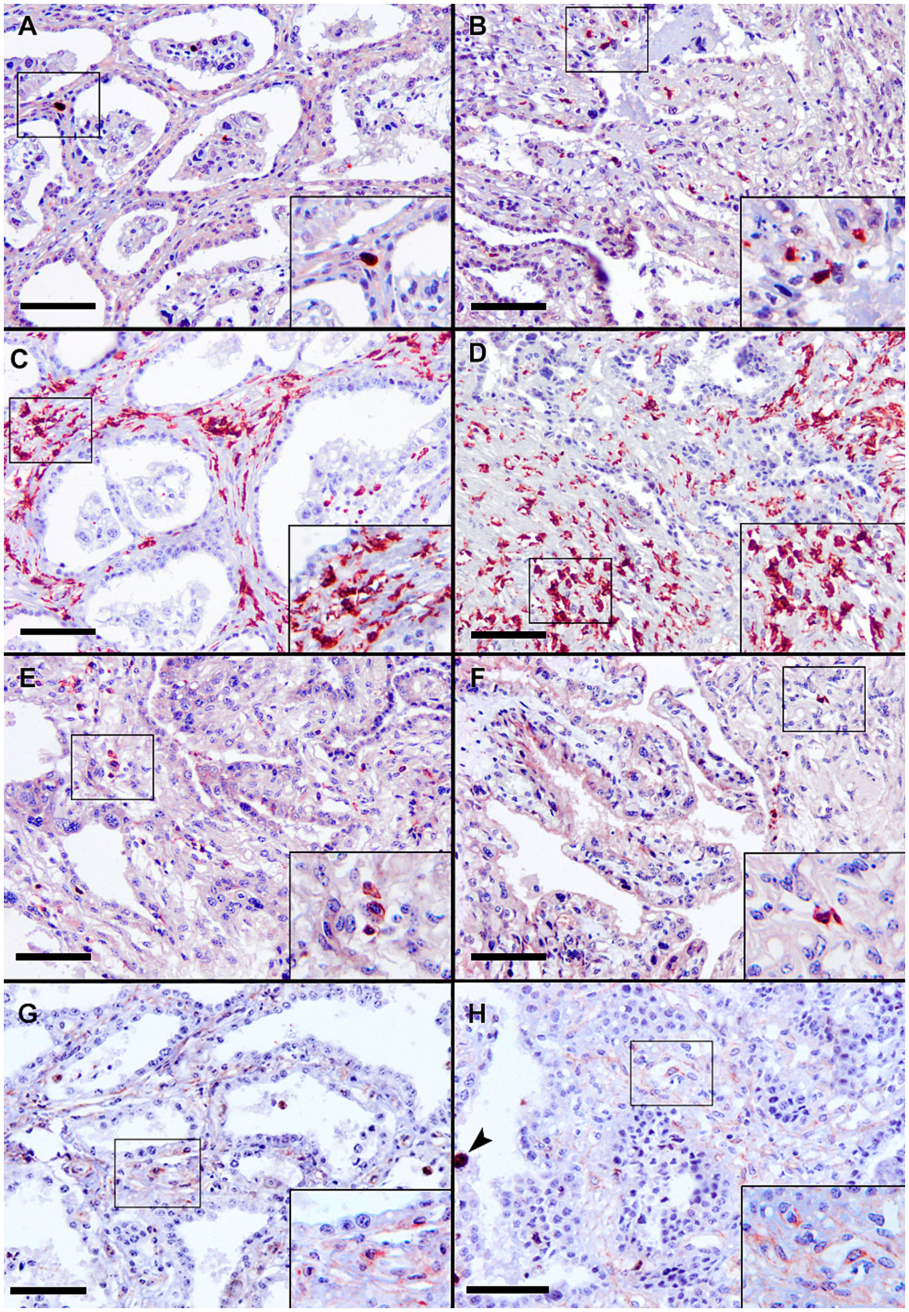
Immunohistochemical labelling in placentas. **(A)** iNOS-positive cells (pro-inflammatory macrophages) in placentas from control dams. **(B)** iNOS-positive cells in BoAHV-1-infected dams. **(C)** CD163-positive cells (anti-inflammatory macrophages) in control placentas. **(D)** CD163-positive cells in infected placentas. **(E)** CD3-positive cells (T lymphocytes) in control placentas. **(F)** CD3-positive cells in infected placentas. **(G)** CD79αcy-positive cells (B lymphocytes) in control placentas. **(H)** CD79αcy-positive cells in infected placentas. Arrowhead indicates immunoreactivity in trophoblastic cells. Insets represent higher magnifications of the fields framed in black. Scale bar = 100 μm.

**Figure 6:**
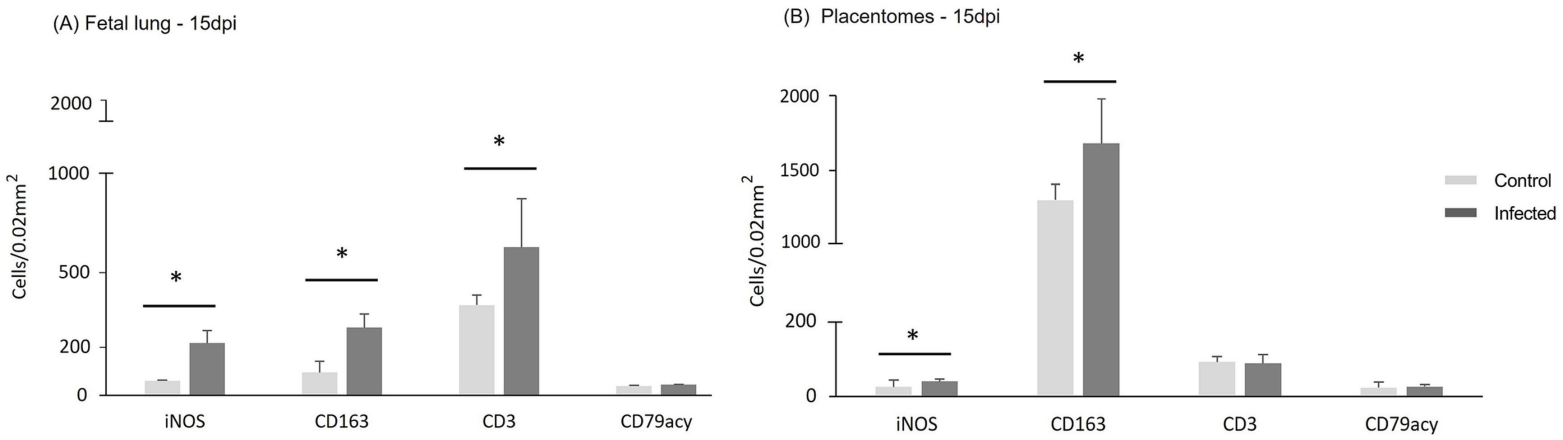
Quantification of immune-cell markers in tissues from control and BoAHV-1-infected animals. **(A)** Fetal lungs. **(B)** Placentomes. Quantitative evaluation (mean ± standard error) of iNOS+, CD163+, CD3 + and CD79αcy + −expressing cells. Statistically significant differences (*p <* 0.05) between control and infected animals are indicated by *.

Immunolabeling patterns for each immune cell population were broadly comparable between control and BoAHV-1-infected animals across fetal lung and placental tissues, although the abundance of positive cells varied markedly depending on the marker assessed (Figures 4–6).

Macrophages expressing iNOS^+^ were detected in both control and BoAHV-1-infected animals, showing granular cytoplasmic staining within diffuse infiltrates of epithelioid cells in the pulmonary parenchyma (Figure 4) and in placenta, including trophoblasts, fetal villi, and caruncular stroma (Figure 5). The number of iNOS-positive cells was significantly higher in infected animals in both organs (*p <* 0.0005) (Figure 6), with greater densities observed in fetal lung tissues (Figure 6).

Macrophages expressing CD163^+^ showed granular cytoplasmic labeling diffusely distributed in the fetal lung parenchyma (Figure 4) and placenta (Figure 5) in both groups. Their abundance was significantly higher in the infected group in both tissues (lung: *p <* 0.0001; placenta: *p* = 0.003) (Figure 6). In contrast to iNOS-positive cells, CD163-positive cells were more numerous in placental tissues, particularly within fetal villi and the caruncular stroma (Figure 6).

T lymphocytes (CD3^+^), characterized by membrane-associated staining, were predominantly found in the lung parenchyma (Figure 4), with lesser detection in the foetal villi and caruncular stroma of the placenta in both groups (Figure 5). CD3 was the main immune marker in fetal lungs, showing cytoplasmic labeling and a significant increase in infected animals (p = 0.003), which exhibited approximately twice the number of T cells compared with controls (Figure 6).

B lymphocytes (CD79*α*cy^+^) exhibited a cytoplasmic staining pattern and were the least abundant immune cell population in lung and placenta in control and infected cows (Figure 6). They were present at low numbers in both tissues, with positive cells located in the pulmonary parenchyma and in the caruncular stroma of the placenta (Figure 5) and no significant differences were observed between groups. Immunoreactivity was also observed in placental trophoblastic cells (Figure 5).

## 4 Discussion

In cattle, late gestation represents a distinct immunological stage characterized by established maternal-fetal tolerance and increased fetal immune maturity (4). At this stage, the bovine fetus is capable of mounting specific cell-mediated and humoral immune responses (5). Within this context, this study evaluated the relative gene expression of endosomal TLRs, cytokines and *BMAP28*, and the presence of innate and adaptive immune cells in distinct fetal and maternal tissues, to characterize immune-associated responses across maternal, placental and fetal compartments following BoAHV-1 transplacental infection. Additionally, endosomal TLR and cytokine expression were assessed in PBLs from infected dams to evaluate the early systemic innate immune response.

The relative expression patterns observed following BoAHV-1 vertical infection differed among maternal, placental and fetal tissues. These findings should be interpreted in the context of the experimental timeline, in which dams were infected at late gestation (day 270) and tissues were evaluated at 15 dpi, a stage at which viral DNA had been detected in selected placental and fetal tissues (22). Because viral DNA was detected only in a limited subset of placental and fetal tissues, additional subgroup analyses comparing PCR-positive and PCR-negative samples were considered statistically underpowered for robust interpretation. In maternal tissues, both PBLs and placenta showed reduced mRNA expression of *TLR9* and *TNF-α*. Previous studies have suggested interactions between BoAHV-1 and NF-κB-associated signaling pathways involved in TLR-mediated responses (17). In this context, the reduced transcript abundance observed in maternal tissues may be consistent with changes in innate immune signaling; however, the present data do not allow direct inference of specific mechanisms such as viral interference with defined signaling pathways or impaired viral sensing. In contrast, fetal tissues infected with BoAHV-1 displayed tissue-specific patterns of transcriptional responses.

The fetal lungs showed increased expression of multiple innate immune-related genes to BoAHV-1 infection, with increased gene expression of all endosomal TLRs, as well as elevated levels of acute inflammatory cytokines and immune mediators, including *TNF-α* and *BMAP28*. Burucúa et al., (28) reported that in BoAHV-1-infected calves, *BMAP28* was downregulated in retropharyngeal lymph nodes, but the expression remained unchanged in nasal mucosa or lungs. This modulation of *BMAP28* aligned with an increased *TNF-α* mRNA expression in tracheal tissue from acutely infected calves (28). The findings in the fetal lungs suggest localized changes in innate immune-associated transcript abundance following BoAHV-1 exposure. Similar patterns have been reported in experimental influenza infection during pregnancy, where infection increases pulmonary inflammatory cytokines and chemokines (29, 30). However, these comparisons should be interpreted with caution due to differences in species, gestational stage, and infection models.

The response in the fetal spleen, a key fetal immune organ, was more specific, with induction of *TLR7* and *TNF-α* and slight *IL-12* reduction. In contrast, the fetal liver presented a distinct transcriptional profile; although *TLR7* and *TLR9* were up-regulated, *TNF-α* and *IL-12* were markedly reduced. Previous studies have reported high viral loads and characteristic lesions in the fetal liver during BoAHV-1 infection (23, 31), supporting its relevance as a target organ in this context.

Placental tissues infected by BoAHV-1 exhibited reduced expression of selected innate immune transcripts, including *TLR9* and *TNF-α* across cotyledons, caruncles and placentomes. These findings are consistent with the tightly regulated immune environment of the bovine placenta, which must balance tolerance to fetal antigens with capacity to respond to pathogens (7). Some studies in other models of vertical infection have reported alterations in placental innate immune responses. For example, Pseudorabies virus (PRV) has been reported to induce TLR up-regulation in murine placentas and embryos (32), whereas *Neospora caninum* infections in pregnant sheep produce mild or absent placental TLR activation depending on the gestational age (33). However, these comparisons should be interpreted cautiously given the biological differences between species and placental structures.

Despite minimal histopathological changes induced by transplacental infection of BoAHV-1 at 15 dpi, as previously reported (22), differences in immune-cell marker labeling were observed between fetal lungs and placental tissues. The fetal lungs showed predominant labeling of CD3^+^ cells and iNOS-associated cells, whereas placental tissues showed a distinct profile with higher representation of CD163-associated cells. These findings indicate tissue-specific differences in immune-cell marker distribution following BoAHV-1 vertical infection at 15 dpi. CD79αcy^+^ cells were consistently scarce in both organs regardless of experimental group. Immunoreactivity was also observed in placental trophoblastic cells, a finding previously reported for this antibody in ruminant placentas (34) and therefore not interpreted as indicative of B lymphocyte infiltration.

Expression of iNOS in fetal lungs is consistent with the presence of immune-cell populations associated with inflammatory-related responses (21, 35). These findings are in line with previous reports describing increased iNOS, TNF-α and COX-2 protein expression in BoAHV-1-infected fetal lungs (22). In the placenta, CD163 expression is commonly associated with macrophage populations linked to anti-inflammatory-related profiles (36, 37). Similar macrophage-associated profiles have been described in bovine placentation and other abortigenic infections, where these cells are considered to participate in processes related to fetal tolerance and tissue remodeling (38). Studies in *N. caninum* infection also show that the placenta mounts an immune response that controls pathogen replication in maternal tissues but not in fetal tissues (33).

Overall, the present findings describe distinct immune-associated response patterns across maternal, placental and fetal tissues following BoAHV-1 transplacental infection in late gestation. Differences in transcript abundance and immune-cell marker distribution were observed between compartments, highlighting variability among tissues at the evaluated time point.

These observations correspond to a single terminal time point (15 dpi) for placental and fetal tissues and should be interpreted accordingly. In this context, transcript abundance represents a dynamic and time-dependent measure of immune activity; therefore, the results are best understood as a snapshot of immune-associated responses rather than a comprehensive characterization of their temporal dynamics, particularly for low-abundance transcripts. Although fetal brain tissue was included in the histopathological examination, no remarkable microscopic lesions were identified at the evaluated time point. Therefore, molecular and immunohistochemical analyses were focused on placental tissues and selected fetal organs relevant to BoAHV-1 pathogenesis. Future studies evaluating the fetal nervous system may contribute to a better understanding of the neuropathogenesis associated with transplacental BoAHV-1 infection. While maternal PBLs were evaluated at earlier time points, the present study does not provide a temporal assessment of tissue-level immune responses or their progression following infection.

In addition, immunohistochemical analyses were performed on a subset of tissues selected based on histological features. Although this approach enabled the evaluation of immune-cell marker distribution in areas with detectable changes, it may introduce selection bias and limits the generalization of these findings across all tissues.

The number of fetuses analyzed in this study was limited, and therefore the findings should be interpreted within the exploratory scope of the study. Nevertheless, the use of a natural-host experimental model provides relevant biological context for the evaluation of immune-associated responses during BoAHV-1 infection in the conceptus.

Complementary approaches, including protein-level expression analyses such as Western blot or additional immunodetection methods, would provide additional support and biological context for these observations. However, the limited availability of tissue material obtained from this large-animal experimental model restricted the incorporation of these complementary analyses for all evaluated targets. Accordingly, the present findings should be interpreted primarily as descriptive immune-associated profiles observed at a single experimental time point. Furthermore, future studies incorporating longitudinal sampling, additional gestational stages and evaluation of viral gene expression in fetal tissues would help to better characterize the temporal dynamics and pathogenesis of maternal and fetal immune-associated responses during BoAHV-1 transplacental infection.

## Data Availability

The original contributions presented in the study are included in the article/Supplementary material, further inquiries can be directed to the corresponding author.
